# DCMD: Distance-based classification using mixture distributions on microbiome data

**DOI:** 10.1371/journal.pcbi.1008799

**Published:** 2021-03-12

**Authors:** Konstantin Shestopaloff, Mei Dong, Fan Gao, Wei Xu

**Affiliations:** 1 Dalla Lana School of Public Health, University of Toronto, Toronto, Ontario, CANADA; 2 Princess Margaret Cancer Centre, University Health Network, Toronto, Ontario, CANADA; University of Washington, UNITED STATES

## Abstract

Current advances in next-generation sequencing techniques have allowed researchers to conduct comprehensive research on the microbiome and human diseases, with recent studies identifying associations between the human microbiome and health outcomes for a number of chronic conditions. However, microbiome data structure, characterized by sparsity and skewness, presents challenges to building effective classifiers. To address this, we present an innovative approach for distance-based classification using mixture distributions (DCMD). The method aims to improve classification performance using microbiome community data, where the predictors are composed of sparse and heterogeneous count data. This approach models the inherent uncertainty in sparse counts by estimating a mixture distribution for the sample data and representing each observation as a distribution, conditional on observed counts and the estimated mixture, which are then used as inputs for distance-based classification. The method is implemented into a *k*-means classification and *k*-nearest neighbours framework. We develop two distance metrics that produce optimal results. The performance of the model is assessed using simulated and human microbiome study data, with results compared against a number of existing machine learning and distance-based classification approaches. The proposed method is competitive when compared to the other machine learning approaches, and shows a clear improvement over commonly used distance-based classifiers, underscoring the importance of modelling sparsity for achieving optimal results. The range of applicability and robustness make the proposed method a viable alternative for classification using sparse microbiome count data. The source code is available at *https*:*//github*.*com/kshestop/DCMD* for academic use.

## Author summary

The uneven performance of conventional distanced-based classifiers when using microbiome profiles to predict disease status has motivated us to develop a novel distance-based method that accounts for uncertainty when modeling sparse counts. We propose a classification algorithm that uses mixture distributions to measure normed distances between microbiome distributions, which better models the underlying structure by handling excess zeros and sparsity inherent in microbial abundance counts. Applications of DCMD have shown improved classification performance and robustness, making the proposed method an improved alternative for classification using microbiome data.

This is a *PLOS Computational Biology* Methods paper.

## Introduction

The increasing accessibility of high-throughput technology has generated a wide array of data types for analysis. One type of data that has recently gained popularity is the microbiome community data, which is composed of site-specific counts for identified bacteria. There is a steadily growing number of studies that have demonstrated associations between the human microbiome and health outcomes, such as inflammatory bowel disease [1], type 2 diabetes [2], and cardiovascular disease [3], making it an important topic of research. However, the presence of sparsity and skewness, which characterizes this type of data, brings a number of challenges to statistical modelling. These challenges have motivated methodological developments that expand the existing algorithms, particularly for classification tasks related to disease risks.

One popular class of approaches often used with microbiome data are distance-based methods, which differentiate and classify samples using distances derived from multivariate measures. Ubiquitous methods include *k*-means [4] and *k*-Nearest Neighbours (*k*-NN) [5], which have been adapted to such data with variable transformations using Euclidean distances, Manhattan distance, and several other measures [6–8]. Other adaptations such as the distance-based nearest shrunken centroid (NSC) classifier, which was developed for the use of microarray data [9]. NSC takes the average of the relative abundances for each class as class centroids [10,11] and then calculate standardized squared distances between new samples and class centroids.

A number of linear and additive machine learning classifiers; such as LASSO, ridge regression (RR), random forest (RF), gradient boosting (GB), and support vector machines (SVM) are also commonly used for high-throughput data [7,11–13]. Some methods rely on penalization (LASSO and RR) in logistic models [14,15], typically with log-transformed adjusted counts or relative abundances of operational taxonomic units (OTUs) to address skewness [16]. The RF and GB algorithms rely on sequentially constructed classifiers and automatically incorporate feature selection [17,18]. Other recent methodological developments for microbiome data include regression models with a phylogenetic tree-guided penalty term [19] and inverse regression to deal with the over-dispersion of zeros in count data [20]. However, the tree-guided method can be overly influenced by tree information [19], and the phylogenetic tree is not always available. The existing methods incorporate observed count data or relative abundance directly when computing distances or defining covariates, with some kinds of transformation of OTUs to account for skewness. None of the methods explicitly account for and model the underlying uncertainty inherent in sparse count data.

This paper aims to address these problems in a classification framework, where predictors are sparse and heterogeneous count data. Shestopaloff et al. [21] proposed representing count data using a mixture distribution to analyze the differences between microbiome communities. We extend the method to distance-based classification using mixture distributions (DCMD) that specifically addresses the uncertainty in sparse and low-count data. DCMD measures the distance between the sample-specific distributions of OTUs rather than between counts or relative abundances, which better models the structure of microbiome data for the distance measure. DCMD is also able to handle excess zero counts, which can potentially improve the predictive accuracy when using sparse OTUs. In this paper, we use two simulation studies to show the advantage of DCMD for classification over existing distance metrics and compare it against common machine learning methods. We provide a comprehensive comparison of distance-based classification methods (*k*-means, *k*-NN, and NSC) and machine learning methods (RF, GB, LASSO, RR, and SVM) in different simulation settings, which to our knowledge has not been studied before. We also illustrate the effectiveness of DCMD on two human microbiome studies [22,23]. The paper concludes with a discussion of the merits, drawbacks and the scope of applicability of the proposed methodology.

## Method

In this section we outline the framework of DCMD. The main steps of the model include mixture distribution specification and parameter estimation for modelling observed data, calculation of conditional distributions for each sample, and calculating distances between samples and cluster centres to use in distance-based classification methods. The mixture model and conditional distribution estimation are described in Shestopaloff et al. [21]. It is proposed to model the underlying population rate structure of the observed count data using a mixture distribution with Poisson-Gamma components, then conditioning on observed sample counts and resolution to obtain sample-specific distributions. In the next step, we use the sample-specific distributions for classification by calculating the distances between distributions.

### Model specification and estimation

Microbiome data typically consists of OTU counts, as illustrated in Table 1. The notations used in our method formulation are as follows:

*n*_*ij*_, *i* = 1,…,*I* for *j* = 1,…,*J*, the count of the *j*th OTU of the *i*th sample.

*N*_*i*_, the total number of aligned reads of sample *i*, *N*_*i*_ = ∑_*j*_*n*_*ij*_

**Table 1:** An OTU table for microbiome data.

	OTU 1	…	OTU J	Total Reads
Sample 1	*n*_11_	**…**	*n*_1*j*_	*N*_1_
⁝	**⁝**		**⁝**	
Sample I	*n*_*I*1_	**…**	*n*_*IJ*_	*N*_*I*_

Without loss of generality, we focus on a specific OTU and omit the *j*th subscript for subsequent notation. Assume that the observed counts, *n*_*i*_, are Poisson distributed with rate *r*_*i*_ = *q*_*i*_*N*_*i*_, *i* = 1,…,*I* for sample *i*, where *q*_*i*_ is the individual-specific relative abundance and is sampled from some general OTU relative abundance distribution *G*_*q*_. Then we have,

$$r_{i}=q_{i}N_{i}=r_{i}^{*}t_{i},$$

where $t_{i}=N_{i}/\bar{N},r_{i}^{*}=q_{i}\bar{N}$, and $\bar{N}=\sum_{i}N_{i}/I$, with $r_{i}^{*}$ sampled from $G=G_{q}\bar{N}$, which is the rate normalized to the average sample reads to make sure that the counts are treated on the same scale. Thus, the observed count for a specific site of OTU is

$$n_{i}|t_{i},r_{i}^{*}\sim Poisson(r_{i}^{*}t_{i}),$$

$$r_{i}^{*}∼G.$$

Since the distribution of OTU is zero-inflated, skewed, and heavy tailed, we propose a mixture distribution to approximate *G*. For positive rates on a given interval, we specify a set of Gamma components, Γ(*α*, *β*), with shape *α* and rate *β*, to cover the range of the data. To separate structural zeros from low-rate and undetected samples, we include a zero-point mass, $n_{i}|t_{i},r_{i}^{*}∼0$, where *P*(*n*_*i*_ = 0) = 1. Additionally, for sparse high rates, we define a high-count point mass, $n_{i}|t_{i},r_{i}^{*}∼C\cdot 1(n_{i}>C)$, where *P*(*n*_*i*_>*C*) = 1, *C* is the truncation point and **1**(∙) is the indicator function. The full set of mixture components is **Ω** = (*G*_*z*_, *G*_1_, *G*_2_,…,*G*_*M*_, *G*_*C*+_) where *G*_*z*_ is a zero-point mass, *G*_*m*_, *m* = 1,2,…,*M*, is a set of Gammas components Γ(*α*_*m*_, *β*_*m*_), and *G*_*C*+_ is a high-count point mass. The process for defining the mixture model components is described in detail in the Simulation section.

Define the weight of each component as

$$w={\left(w_{z},w_{1},w_{2},\ldots ,w_{M},w_{C+}\right)}^{\prime },$$

where *w*_*z*_ is the weight of the zero-point mass, *w*_*m*_, *m* = 1,2,…,*M*, is the weight for *m*th corresponding Gamma component, and *w*_*C*+_ is the weight of high-count point mass. Define

$$y_{x}=\sum_{i}{1(n}_{i}=x),$$

the number of species observed *x* times across all samples for *x* = *z*, 0,1,2,…,*C*,*C*+. Then our goal is to minimize $\sum_{x=0}^{C+}{\left[y_{x}-y_{x}^{E}\right]}^{2}$, where $y_{x}^{E}$ is the expected aggregate counts of *y*_*x*_. Note that given Γ(*α*_*m*_, *β*_*m*_), sample counts conditional on *t*_*i*_ are distributed as a negative binomial *NB*[*α*_*m*_, *β*_*m*_/(*t*_*i*_+*β*_*m*_)] [21]. Define

$$p_{xmi}=P_{NB}(X=x|t_{i},\alpha_{m},\beta_{m})$$

as the probability of observing count x from the mth mixture component conditional on the resolution *t*_*i*_. Then $y_{x}^{E}=\sum_{w_{m}\in w}w_{m}p_{xm}∙I$, where *p*_*xm*_ = ∑_*i*_*p*_*xmi*_/*I*. Thus, we have the objective function:

$$\arg\;\underset{\vec{w}}{\min}\sum_{x=z}^{C+}{\left[y_{x}-\left(\sum_{w_{m}\in w}w_{m}p_{xm}\right),I\right]}^{2},$$ (1)

$$s.t.\sum_{m}w_{m}=1,w_{m}\geq 0,\forall m.$$

The estimate, $\hat{w}$, is obtained by optimizing the least-squares objective function (1), using the Broyden-Fletcher-Goldfarb-Shanno (BFGS) algorithm [24] with the augmented Lagrangian method [25] for the constraints.

Due to the sparse nature of the data, we only optimize the weights and fix the Gamma parameters. Attempting to model the low-rate structure by optimizing both weights and Gamma parameters (*α*_*m*_, *β*_*m*_) via expectation-maximization (EM) results in biased structural zero estimates and a poor overall fit of the low counts [26]. In this context, the EM is also prone to numerical issues, convergence to local minima and can often be too slow computationally for this type of application [26]. On the other hand, BFGS provides a much faster and robust alternative.

### Weighted mixture distribution

To address the uncertainty around specifying components for the mixture model, particularly for the low rates where sparsity is often an issue, we define a set of nested models Φ_*l*_, *l* = 1,…,*L*, with varying components for modelling the rate structure around zero. We estimate the joint mixture model using a nonparametric bootstrap algorithm. As stated in Shestopaloff et al. [21], we can obtain the weight *v*(*l*) of each candidate model, which is the proportion of times each model is selected as optimal relative to the observed data, and calculate the weights for the joint mixture distribution. Let ***w***_*l*_ be the estimated weights for each candidate model, Φ_*l*_, with zeros assigned to the weights of components not included in a specific model, then the weights of the joint model are ***w*** = ∑_*l*_*v*(*l*)***w***_*l*_.

### Sample-specific distribution

Once we have a distribution for the OTU, we can estimate sample-specific distributions by conditioning on the observed count *n*_*i*_, estimated mixture weights ***w***, and resolution *t*_*i*_. We can obtain the probability that sample *i* sampled from a specific component, as:

$$p_{im}=P\left(i\in G_{m}|n_{i},t_{i},w\right)=w_{G_{m}}\frac{\Gamma \left(n_{i}+\alpha_{m}\right)}{\Gamma \left(n_{i}+1\right)\Gamma \left(\alpha_{m}\right)}{\left(\frac{\beta_{m}}{t_{i}+\beta_{m}}\right)}^{\alpha_{m}}{\left(1-\frac{\beta_{m}}{t_{i}+\beta_{m}}\right)}^{n_{i}}.$$ (2)

The probability of being assigned to the zero-point mass is *P*(*i*∈*G*_0_) = **1**(*n*_*i*_ = 0) and to the high-count point mass is *P*(*i*∈*G*_*C*+_) = **1**(*n*_*i*_>*C*). Define the sample-specific mixture weights as

$$w_{i}={\left(w_{iz},w_{i1},\ldots ,w_{iC},w_{iC+}\right)}^{\prime },$$

where

$$w_{im}=P(i\in G_{m})/\sum_{m}P(i\in G_{m})=p_{im}/\sum_{m}p_{im}.$$

Since the sample-specific weights have been adjusted for the individual resolutions *t*_*i*_ through the *p*_*im*_ probabilities, the Poisson-Gamma mixture probabilities are *NB*(*α*, *β*/(1+*β*)). Also note that we have differentiated the zeros in our mixture distribution, which are defined as structural zeros, *x = z*, and observed zeros, *x =* 0. Given the underlying rate distribution from the joint mixture model, we can then calculate the probability of observing count *x* = *z*, 0,1,…,*C*, *C*+ from each mixture component *G*_*m*_ as

$$P_{G_{m}}(x)=P(X=x|G_{m})=P_{NB}(X=x\alpha_{m},\beta_{m}).$$

For the point masses we have *P*(*X* = *x*|*G*_*z*_) = **1**(*n*_*i*_ = 0) and *P*(*X* = *x*|*G*_*C*+_) = **1**(*n*_*i*_>*C*), respectively. To simplify the representation of the distribution, define a vector of probabilities

$$P(x)=[P(X=x|G_{z}),P(X=x|G_{1}),\ldots ,P(X=x|G_{m}),P(X=x|G_{C+})]$$

$$=[P_{G_{z}}(x),P_{G_{1}}(x),\ldots ,P_{G_{M}}(x),P_{G_{C+}}(x)],$$

for *x* = *z*, 0,1,…,*C*, *C*+. Then we can define the discrete probability density for sample *i* as

$$P_{i}=[P_{i}(z),P_{i}(0),\ldots ,P_{i}(C),P_{i}(C+)]={w_{i}}^{\prime }P,$$

where

$$P_{i}(z)=w_{G_{z}},$$

$$P_{i}(C+)=1-[\sum_{x=0}^{C}P_{i}(x)+P_{i}(z)],$$

P=[P(z),P(0),…,P(C),P(C+)].

**Fig 1:**
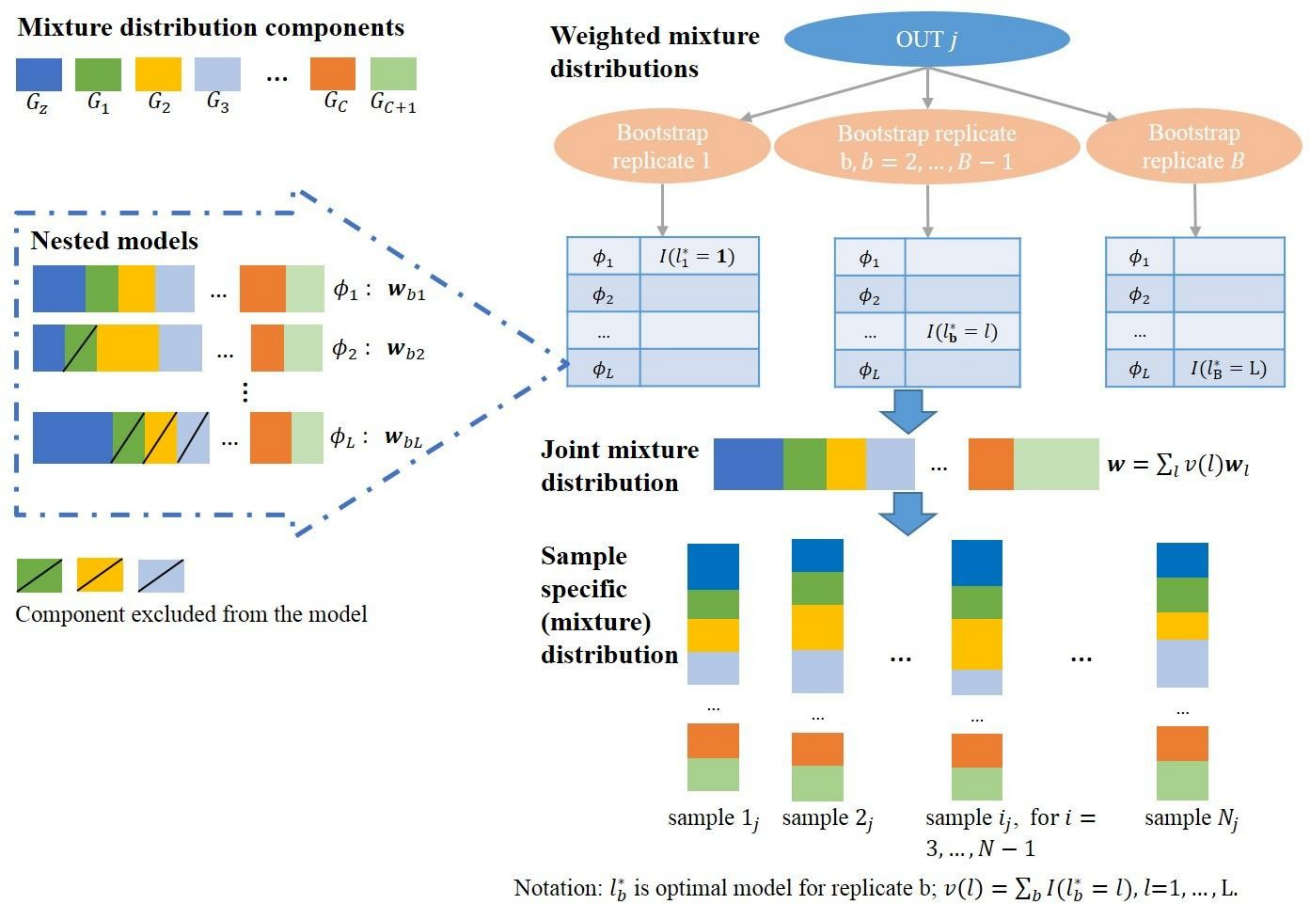
Workflow for obtaining a sample-specific mixture distribution for each sample i in OTU j: 1) Specify a set of nested candidate mixture distributions using a specific set of components; 2) Apply bootstrap to the set of nested models and calculate the weights of each candidate mixture model, then calculate the weights of the joint mixture distribution; 3) Estimate sample-specific distributions conditional on *n*_*i*_, *t*_*i*_, and the joint mixture distribution.

The ***P***(*x*) vectors in the matrix ***P*** are the vectors giving the probability of observing *x* from each mixture component, which can be pre-calculated for distance calculations. The overview of how to obtain sample-specific mixture distributions given a set of mixture distribution components is shown in Fig 1.

### Classification

Once the distribution for each sample has been computed, we use *k-*means and *k-*Nearest Neighbours (*k*-NN) algorithms for classification. In this section, we outline how to apply these algorithms using two distance measures, discrete *L*^2^ (D-*L*^2^) norm and continuous cumulative *L*^2^ (CC-*L*^2^) norm.

### Distance measures

Given posterior probability *f*_*i*_, cumulative posterior probability *F*_*i*_, and an estimated set of weights *w*_*i*_ for sample *i*, the distance metrics are:

**D-*L*^2^ Norm**:

$$\delta_{D-L^{2}}(f_{i},f_{j})=\sum_{x}{\left[f_{i}(x)-f_{j}(x)\right]}^{2}$$

$$=\sum_{x}{\left[(w_{i}-w_{j})P\left(x\right)\right]}^{2},$$ (3)

where *x* = *z*, 0,1,…,*C*, *C*+. Note that we include the structural zero component, z, separately and that the distances only depend on the weights. For multiple predictors, j = 1, …, J, the total distance between samples *i*_1_ and *i*_2_ is the sum across all predictors, $D(i_{1},i_{2})=\sum_{j}\delta_{j}(f_{i_{1}},f_{i_{2}})$.

**CC-*L***^**2**^ **Norm**:

$$\delta_{CC-L^{2}}(F_{i},F_{j})=\int_{0}^{C}{\left[F_{i}(x)-F_{j}(x)\right]}^{2}dx$$

$$=\left(w_{i}-w_{j}\right)G_{m_{1}m_{2}}{\left(w_{i}-w_{j}\right)}^{\prime },$$ (4)

where $G_{m_{1}m_{2}}$ is a matrix with the (*m*_1_, *m*_2_) entry set to $\int G_{m_{1}}(x)G_{m_{2}}(x)dx$ for each of the continuous mixture component. Details of the derivation can be found in Shestopaloff [26].

#### Distance-based classification

We use the distances calculated in Eqs (3) and (4) in a *k*-means and *k*-NN framework. In *k*-means, the mean of each class is calculated from the training data and points are classified to the nearest class. In *k*-NN, samples are classified as the mode of the labels from *k* closest neighbours of the training set. The steps of *k*-means and *k*-NN algorithms are as follows:

***K*-means**: To adapt the *k*-means algorithm, we estimate the mean distribution for each class by minimizing the distributional distances between it and the class samples, conditional on a specified distance. Since distances are *L*^2^ norms and only depend on the weights, as shown in Eqs (3) and (4), the mean of the weights for each class gives the optimum. The algorithm is implemented as follows:

Step 1: Determine the mean of the weights for the *j*th predictor in class *k*,

$w_{\mu_{k},j}=\sum_{i\in k,j}w_{k,j}/|N_{k,j}|$, where |*N*_*k*,*j*_| is the number of samples in class *k* of predictor j, *k* = 1,…,*K* and *j* = 1,…,*J*;

Step 2: Compute the distance to the mean for sample *i* across all predictors,

$$D(i,\mu_{k})=\sum_{j}\delta (P_{i,j},P_{\mu_{k},j});$$

Step 3: Predict the label of sample *i* as the closest mean,

$${\hat{y}}_{i}=\underset{k}{\mathrm{argmin}}\;D(i,\mu_{k}).$$

***K*-NN:** After computing the pairwise distances between samples and summing across predictors, these can be used directly to identify the nearest neighbours for classification. The algorithm for *k*-NN is as follows:

Step 1: Compute the pairwise distance of sample *i*_1_ and *i*_2_, *i*_1_, *i*_2_ = 1,…,*I*, *i*_1_≠*i*_2_,

$$D(i_{1},i_{2})=\sum_{j}\delta (P_{i_{1},j},P_{i_{2},j});$$

Step 2: For sample i, pick the k samples with smallest distance to sample *i*, the optimal *k* can be determined using cross-validation (CV) in the training set or existing heuristics.
Step 3: Tally the labels of the k nearest neighbours, then sample i is predicted as the mode of the *k* labels.

**Fig 2:**
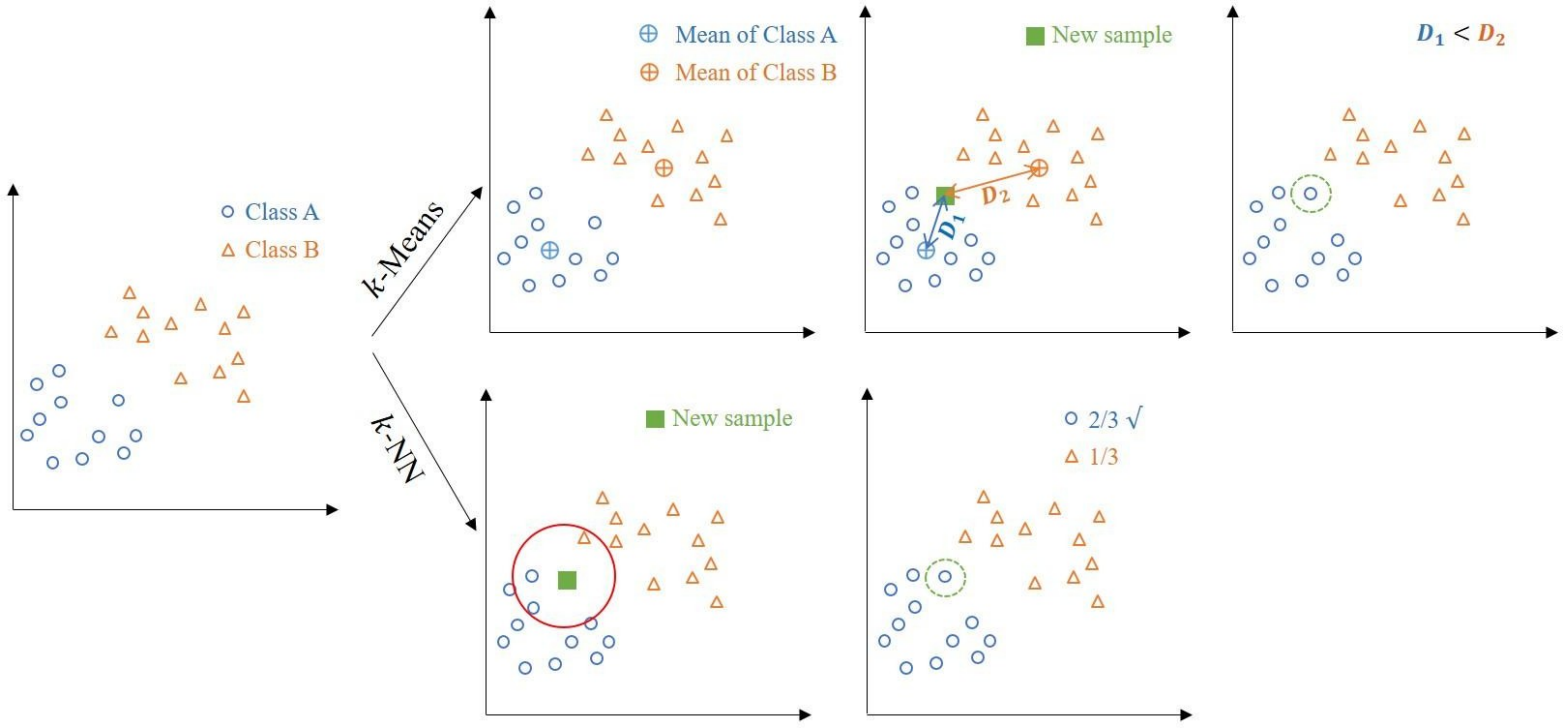
Illustration of k-means and k-NN framework using sample-specific distributions. For k-means (top panel), the distance between the new sample to the mean of class A is smaller than to the mean of class B, hence the new sample is predicted as class A. For k-NN (bottom panel), using 3 nearest neighbours, the new sample is predicted as class A.

The overall workflow of DCMD within the *k*-means and *k*-NN frameworks is presented in Fig 2.

### Predictive metrics

Let ${\hat{y}}_{i}$ be the predicted class for sample *i*. The classification accuracy is defined as the proportion of correctly predicted cases: $\mathrm{Accuracy}=\frac{1}{I}\sum_{i=1}^{I}I({\hat{y}}_{i}=y_{i})$. For binary outcomes we also include precision, recall, and F1 score as metrics to measure predictive performance. We count the number of true positive (*TP*), false positive (*FP*), and false negative (*FN*) and defined these metrics as follows [27]:

Precision=TP/(TP+FP),

Recall=TP/(TP+FN),

F1Score=2×precision×recall/(precision+recall).

## Simulation

### Data generation

To evaluate the performance of the DCMD method, we design simulation studies that mimic microbiome community count data and assess classification performance. We simulate a separate mixture distribution for each class, individual sample rates, and resolutions to generate observed counts. For the mixture distribution, the number of components, M, is sampled from *Unif*(5, 15). And the number of samples to be taken from each mixture component is set by binning samples from a *Beta*(*α*_*b*_, *β*_*b*_) at uniform intervals, with the *α*_*b*_ varied to give different class means and levels of sparsity and with *β*_*b*_~*Unif*(2, 6.5) to control dispersion. The observed counts for each sample are then generated as $n_{i}∼Poisson(r_{i}^{*}t_{i})$, where $r_{i}^{*}$ is the sampled rate and resolution *t*_*i*_~*Unif*(2/3, 5/4).

We consider two- and three-class outcomes with several simulation scenarios for each case. For the two-class outcome, parameter settings and summary statistics for each scenario are shown in Table 2. Scenarios 1 and 2 have low sparsity data, and scenarios 3 and 4 are highly sparse. Scenarios 1 and 3 have weakly differentiated classes (small difference in *α_b_*), while scenarios 2 and 4 have strongly differentiated classes (large difference in *α_b_*). The sample size is *I* = 800, with 400 samples per class and *J* = 25 OTUs. For the three-class outcome, parameter settings and summary statistics are shown in S1 Table. Scenarios 1–3 have strongly differentiated classes, with varying levels of sparsity. The sample size is *I* = 1200, with 400 samples in each class and *J* = 25 OTUs. A null case scenario is also generated by permuting class labels, and performance metrics for each outcome and scenario are computed over 100 simulation replicates.

**Table 2:** Two-class outcome: the parameter settings for each scenario and the corresponding summary statistics of each class over 100 replicates.

Scenario	Signal	Sparsity	Class	Size	*α*_*b*_ range	Mean ZP (SD)	Mean
1	Weak	Low	1	400	(1.5, 1.8)	0.32 (0.13)	7.91
			2	400	(1.8, 2.1)	0.26 (0.12)	9.63
2	Strong	Low	1	400	(1.5, 1.8)	0.33 (0.13)	7.74
			2	400	(2.7, 3.0)	0.14 (0.09)	15.12
3	Weak	High	1	400	(0.2, 0.4)	0.84 (0.07)	1.05
			2	400	(0.4, 0.6)	0.74 (0.10)	1.92
4	Strong	High	1	400	(0.2, 0.4)	0.84 (0.07)	0.98
			2	400	(0.8, 1.0)	0.56 (0.13)	3.61

### Mixture model specification

The specification of the mixture model components should be data-driven, and the main requirement is that the Gammas allow for appropriate coverage of the observed data. We split the count data into five intervals and apply different strategies to specify components on each interval. Modelling of the zeros and low-rate structures is based on [28], and modelling of the higher counts is based on [21].

1. Structural zeros: For data with observed zeros, a zero-point mass P(*X* = 0) = 1 is included to model zero inflation.
2. Low counts (*x*∈[0,1,2,3]): We specify components as Poisson rate posteriors with uniform priors for each of the counts, which is *Γ*(*x*+1, 1). Hence, we include *Γ*(1,1), *Γ*(2,1), *Γ*(3,1) and *Γ*(4,1). The cut-off is set to *x* = 3 because rate posteriors for higher values have a low probability of observing zero, and we want to differentiate the distributions relevant to modelling zero inflation. We also want to examine whether more mass close to zero improves the fit for the low rates. Therefore, we add exponentials with a higher rate, *β*, to have more mass near zero. In this case, we include *Γ*(1,2) into the candidate models. We can potentially include *Γ*(1,3) or other terms into the model, then apply the procedure described above to select the optimal mix. A fuller discussion, drawn from modelling sparse counts for total species estimation, can be found in [28].
3. Integer counts (*x*∈[4,5,6,7]): Components in this range are also specified as the rate posterior *Γ*(*x*+1, 1) at integer intervals. This block exists as a buffer to ensure no gaps in the coverage after the low-count distributions, as this can potentially bias the structural zero and low-rate estimates. This is specified until the last integer component has little overlap with the previous low-rate component. In our formulation, we use an upper limit of *x* = 7 as simulations showed negligible differences between *x* = 7 and *x* = 8. The integer components include *Γ*(4,1),…,*Γ*(7,1).
4. High counts (*x*∈[8,…,*C*]): The higher counts tend to have a large range, and it’s not practical to specify them on integer intervals. In this case, we set the number of components based on the range of the data and specify *α*_*m*_ at uniform intervals on a linear-log scale from 8 to *C* = *q*_*p*_, a set quantile of the data. Using between 10 and 15 components worked well in past applications [21]. For our modelling, p = 0.85 is an effective threshold, which means C is the 85% quantile of the sample.
5. Extreme high counts (*x*>C): These counts are truncated to a point mass P(*X*>*C*) = 1, in part because of the low density in this range and the uncertainty in modelling them and in part to decrease computation time. The mixture model specification heuristics described above are primarily for modelling low-abundance OTU, which covers the most information of the microbiome data. Higher abundance OTU can be modelled by restricting the component specification to higher counts and increasing p.

The full model we use for our data includes [*Γ*(1,2), *Γ*(1,1),…,*Γ*(7,1), *Γ*(8,1)], along with varying high-count components. Nested models are generated by progressively excluding *Γ*(1,2), [*Γ*(1,2), *Γ*(1,1)],… for a total of five models. A sample model specification for one of the OTU is presented in S2 Table.

### Model fitting and comparison methods

The proposed method is compared with *k*-means and *k*-NN using Euclidean and Manhattan distances of relative abundances, distance-based NSC, as well as LASSO, RF, GB, RF and SVM classifiers [29]. Models are trained using a 60/40 training and test set split [30], with the training set remaining the same for all classifiers within each replicate. For the machine learning methods, we use existing packages and tune the hyper-parameters using cross-validation when appropriate, see details in S1 Text.

### Simulation results

**Fig 3:**
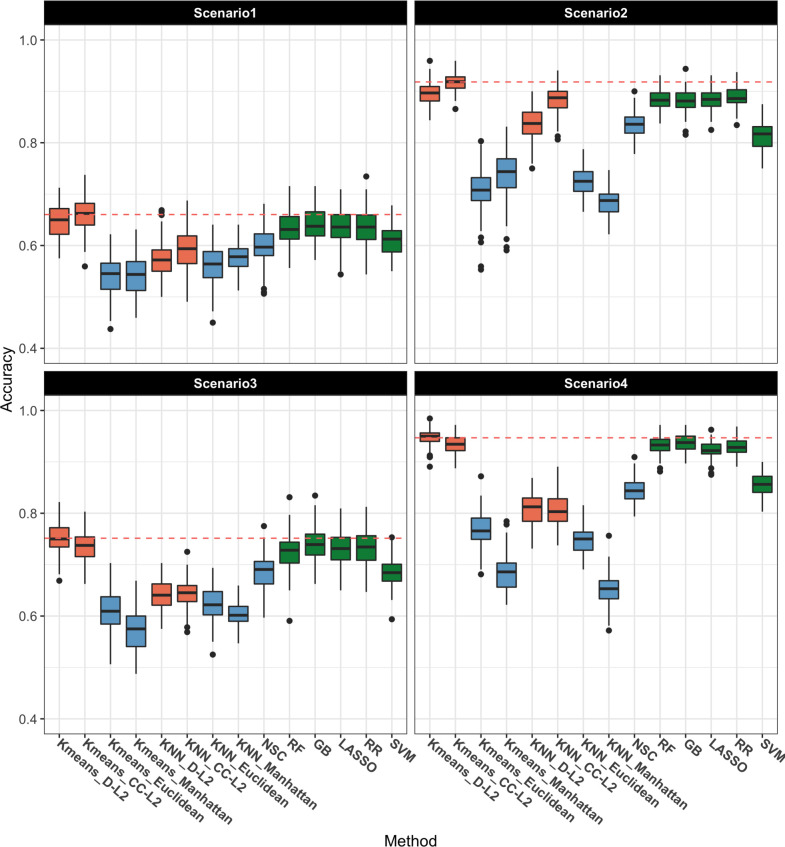
Two-class outcome: boxplot of the accuracy over 100 replicates for each method and scenario. The proposed DCMD method is shown in orange for *k*-means and *k*-NN with D-*L*^2^ and CC-*L*^2^ distances. The other distance-based methods are shown in blue, including *k*-means and *k*-NN with Euclidean and Manhattan distances and NSC. Machine learning methods are shown in green, including random forest (RF), gradient boosting (GB), LASSO, ridge regression (RR), support vector machine (SVM). The dashed red line gives the average accuracy of the best method in each scenario.

For the two-class outcome, classification accuracy for each model and scenario is presented in Fig 3. The orange boxplots are the results for the proposed DCMD method in a *k*-means and *k*-NN framework. The blue boxplots are the other distance-based methods, including *k*-means and *k*-NN with Euclidean and Manhattan distance and NSC. The green boxplots give results for the machine learning methods, including RF, GB, LASSO, RR, and SVM. The dashed red line gives the average accuracy of the best method in each scenario. The results show that in Scenarios 1 and 2, when sparsity is low, *k*-means with *CC-L*^*2*^ norm performs best, followed by *k*-means with D-*L*^2^ norm, while in Scenarios 3 and 4, when sparsity is high, *k*-means with D-*L*^2^ norm gives the best performance, followed by *k*-means with *CC-L*^*2*^ norm. Overall, DCMD in a *k*-means framework with *L*^*2*^ norms outperforms the other classification methods for all types of signals and data structures for the two-class outcome. Differences in accuracy within the distance-based methods are also progressively more pronounced in favour of DCMD, among which *k*-means outperforming *k*-NN. The specialized NSC approach performed similarly to DCMD within the *k*-NN framework. However, NSC generally falls short of *k*-means DCMD and other machine learning methods.

Table 3 shows the summary statistics of the F1 Score over 100 replicates for the two-class outcome. The top results in each scenario are highlighted. Similar to accuracy, DCMD with *L*^*2*^ norms produce the highest F1 Scores (F1 Score (SD) = 0.68 (0.034), 0.92 (0.017), 0.77 (0.028), 0.95 (0.014) in Scenarios 1–4, respectively), which are better than the best machine learning method (GB: 0.64 (0.033), RF and RR: 0.89 (0.019), LASSO: 0.75 (0.030), GB: 0.94 (0.016) in Scenarios 1–4, respectively). DCMD shows consistent good performance in each scenario compared among the methods.

**Table 3:** Two-class outcome: the summary of F1 Scores for each model over 100 replicates.

Model	Scenario 1 (SD)	Scenario 2 (SD)	Scenario 3 (SD)	Scenario 4 (SD)
*k*-means-*D-L*^*2*^	0.67 (0.033)	0.90 (0.019)	**0.77 (0.028)**	**0.95 (0.014)**
*k*-means-*CC-L*^*2*^	**0.68 (0.034)**	**0.92 (0.017)**	0.76 (0.028)	0.94 (0.016)
*k*-means-Euclidean	0.56 (0.054)	0.73 (0.047)	0.66 (0.039)	0.79 (0.026)
*k*-means-Manhattan	0.58 (0.065)	0.78 (0.040)	0.69 (0.030)	0.77 (0.022)
*k*-NN-*D-L*^*2*^	0.59 (0.050)	0.85 (0.028)	0.74 (0.022)	0.85 (0.021)
*k*-NN-*CC-L*^*2*^	0.59 (0.048)	0.88 (0.028)	0.73 (0.025)	0.85 (0.020)
*k*-NN-Euclidean	0.60 (0.043)	0.77 (0.023)	0.68 (0.031)	0.79 (0.020)
*k*-NN-Manhattan	0.66 (0.037)	0.77 (0.015)	0.71 (0.017)	0.75 (0.016)
NSC	0.56 (0.067)	0.85 (0.023)	0.69 (0.046)	0.86 (0.022)
RF	0.63 (0.036)	**0.89 (0.019)**	0.74 (0.038)	0.94 (0.017)
GB	**0.64 (0.033)**	0.89 (0.020)	0.75 (0.033)	**0.94 (0.016)**
LASSO	0.64 (0.039)	0.89 (0.021)	**0.75 (0.030)**	0.93 (0.015)
RR	0.62 (0.052)	**0.89 (0.019)**	0.74 (0.037)	0.93 (0.015)
SVM	0.64 (0.031)	0.82 (0.028)	0.72 (0.026)	0.86 (0.021)

**Fig 4:**
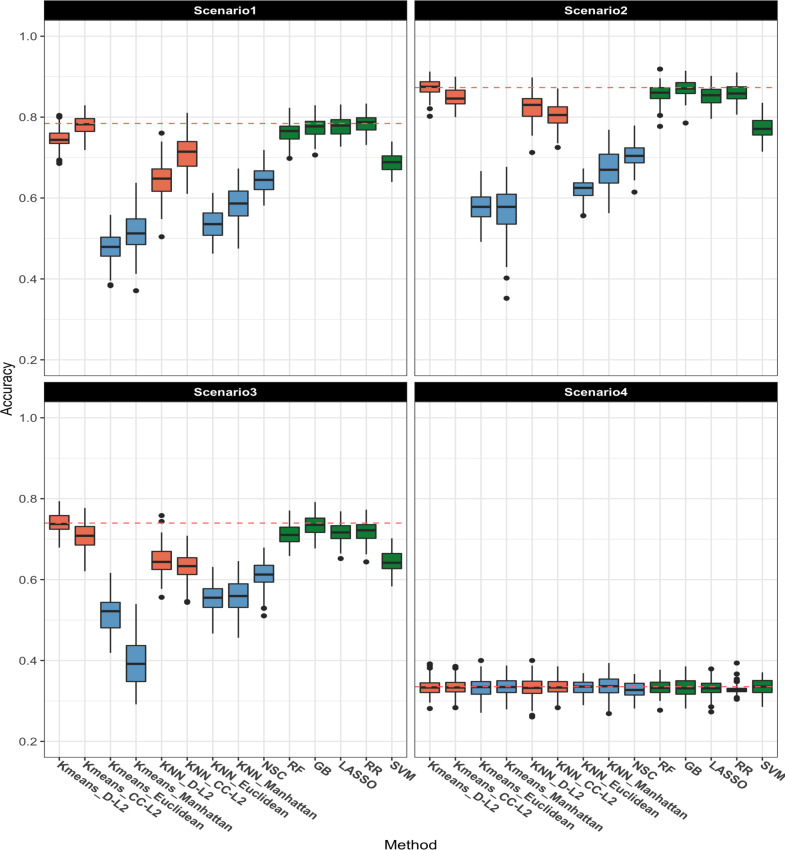
Three-class outcome: boxplot of accuracy over 100 replicates for each method and scenario. The proposed DCMD method is shown in orange for k-means and k-NN with D-L^2^ and CC-L^2^ distances. The other distance-based methods are shown in blue, including k-means and k-NN with Euclidean and Manhattan distances and NSC. Machine learning methods are shown in green, including random forest (RF), gradient boosting (GB), LASSO, ridge regression (RR), support vector machine (SVM). The dashed red line gives the average accuracy of the best method in each scenario.

The classification accuracy of each model and scenario for a three-class outcome is presented in Fig 4. For Scenarios 1–3, the classes are differentiated under varying levels of sparsity, and we observe that DCMD is competitive with the optimal machine learning methods. Although RR has similar predictive accuracy to DCMD in Scenario 1 and 3, and GB has similar predictive accuracy in Scenario 2, DCMD is consistently improved over the optimal comparison method. None of the models is systematically over-fit, as predictive accuracy in the null case (Scenario 4) is near the baseline accuracy of 0.33.

## Application

### Data description

We test our method on data from two microbiome studies. The first is a study on colorectal cancer reported by [22]. A total of 190 samples (95 pairs) were collected from 95 patients in Vall d’Hebron University Hospital in Barcelona and Genomics Collaborative. The study aimed to identify associations between tumor microbiome and colorectal carcinoma. Both the colorectal adenocarcinoma tissue and adjacent non-affected tissues were collected. The OTU count table generated by 16S amplification was obtained from the Microbiome Learning Repo [12]. Prior to model training, eighteen samples with total reads less than 100 were dropped from the dataset, and we also excluded OTUs with mean relative abundance less than 0.001, resulting in 149 OTUs and 172 samples (86 pairs) used to differentiate tumour and normal tissue. The second study is a case-control study of Crohn’s disease (CD) from a multi-center cohort, which was designed to examine how microbiota contributes to CD pathogenesis [23]. The profiles were obtained using Illumina 16S rRNA sequencing. The dataset was downloaded from the Microbiome Learning Repo [12] and consisted of 140 ileal tissue biopsy samples. Minimal sample depth is set at 100, and OTUs are restricted to less than 90% zero proportion, leaving 140 samples (78 cases and 62 controls) and 31 OTUs for analysis.

### Model fitting and evaluation

For both datasets, we compare our proposed *L*^2^-norm based *k*-means and *k*-NN classifier with five other distance-based classifiers (*k*-means-Euclidean, *k*-means-Manhattan, *k*-NN-Euclidean, *k*-NN-Manhattan, NSC) and six machine learning methods (RF, GB, LASSO, RR, SVM). We assess model performance using 10-fold CV. In each iteration, one fold of the data is treated as the test set, and the remaining nine are used for training. The specification of DCMD and other classifiers is the same as that in the simulations (see S1 Text). We calculate accuracy, precision, recall, and F1 score as metrics for comparison.

For the colorectal cancer data, we reduce the predictor space for distance-based classifiers by the univariate screening of OTUs with a nonparametric Mann-Whitney U test on the training set. To adjust for multiple comparisons, we use q-values obtained by the Benjamini–Hochberg (BH) method [31] and retain OTUs with q-values less than 0.05 in each training set. The mean number of OTUs selected from each training set is 42 (range: 13–57). For the machine learning approaches, we include all 149 OTUs. For the CD dataset, 31 OTUs are included for all methods.

### Applied results

The predictive performance of each classifier for the colorectal cancer and CD studies are presented in Tables 4 and 5, respectively. The accuracy of *k*-means with *D-L*^*2*^ norm is 0.67 for colorectal cancer and 0.73 for CD, which is the best method for colorectal cancer and the second-best for CD. The F1 scores are also among the highest for both datasets, indicating that DCMD has consistently optimal performance and an improvement over the other classifiers. Predictive accuracy of *k*-means with *CC-L*^*2*^ norm is slightly worse, likely due to high zero proportions in the predictors, which is consistent with the simulation results. Similarly, DCMD outperforms Euclidean and Manhattan distances within *k*-means, and *k*-means outperforms *k*-NN overall. Within *k*-NN, accuracy and F1 score indicate that DCMD has predictive performance comparable to Euclidean or Manhattan distances. The NSC approach has an accuracy of 0.67 and a precision of 0.72 for colorectal cancer, with a recall of 0.56 and an F1 score of 0.63, notably lower than that of the *k*-means classifiers. The performance is unstable in the CD data.

**Table 4:** Dataset 1—Colorectal Cancer: the predictive performance of the 14 classifiers.

Method	Accuracy	Precision	Recall	F1 score
*k*-means-*D-L*^*2*^	**0.67**	0.66	**0.69**	**0.67**
*k*-means-*CC-L*^*2*^	0.63	0.62	0.67	0.65
*k*-means-Euclidean	0.62	0.62	0.60	0.61
*k*-means-Manhattan	0.65	0.66	0.60	0.63
*k*-NN-*D-L*^*2*^	0.65	**0.77**	0.43	0.55
*k*-NN-*CC-L*^*2*^	0.63	0.65	0.57	0.61
*k*-NN-Euclidean	0.63	0.66	0.55	0.60
*k*-NN-Manhattan	0.61	0.69	0.40	0.50
NSC	**0.67**	**0.72**	0.56	0.63
RF	0.60	0.61	0.59	0.60
GB	0.63	0.63	0.64	0.64
LASSO	0.59	0.59	0.59	0.59
RR	0.64	0.63	**0.69**	**0.66**
SVM	0.66	0.67	0.63	0.65

**Table 5:** Dataset 2—Crohn’s Disease: the predictive performance of the 14 classifiers.

Method	Accuracy	Precision	Recall	F1 score
*k*-means-*D-L*^*2*^	**0.73**	**0.75**	0.78	**0.76**
*k*-means-*CC-L*^*2*^	0.72	**0.75**	0.74	0.75
*k*-means-Euclidean	0.68	0.69	0.76	0.72
*k*-means-Manhattan	0.66	0.67	0.76	0.71
*k*-NN-*D-L*^*2*^	0.62	0.62	**0.85**	0.71
*k*-NN-*CC-L*^*2*^	0.61	0.61	0.83	0.71
*k*-NN-Euclidean	0.65	0.66	0.78	0.71
*k*-NN-Manhattan	0.61	0.62	0.77	0.69
NSC	0.66	0.64	**0.90**	0.74
RF	0.69	0.71	0.76	0.73
GB	0.68	0.69	0.76	0.72
LASSO	**0.74**	**0.76**	0.78	0.73
RR	0.69	0.69	0.78	0.73
SVM	**0.74**	0.74	0.81	**0.77**

Compared to the machine learning methods, DCMD with *k*-means is superior to RF, GB, LASSO, RR, and SVM in the first dataset. When results are replicated controlling for distance-based classifier variable selection (S3 Table), machine learning methods has improved performance, except for GB. In the second dataset, LASSO and SVM are the best methods with accuracies of 0.74, slightly outperforming the accuracy of 0.73 for *k*-means with *D-L*^*2*^ norm. Otherwise, DCMD *k*-means with *D-L*^*2*^ and *CC-L*^*2*^ norms either equivalent or outperform the machine learning approaches.

## Discussion

The results of our simulation studies and microbiome applications indicate that the proposed DCMD method performs well over a range of scenarios, achieving good classification performance when using sparse data as predictors. The predictive accuracy is consistently improved compared to other distances within distance-based classifiers. It is either advantageous or competitive compared to a number of machine learning methods under a wide range of scenarios. The improved performance of DCMD on sparse data results from the use of mixture distributions to represent the observed count data because the mixture distributions can not only model the underlying uncertainty in the observed sample counts but also account for zero inflation. The improvement is particularly significant in comparison to other distances within the regular *k*-means and *k*-NN classifiers.

The performance differences between the *D-L*^*2*^ and *CC-L*^*2*^ norms can be attributed to the data structure. In less sparse scenarios, the data structure is better modelled by a continuous rate structure, resulting in a slight advantage for the *CC-L*^*2*^ metric. While in the higher ZP and low-count scenarios, the *D-L*^*2*^ norm allows us to use specific differentiation of zeros into structural and non-structural and modelling expected counts directly, which can further capture the general structure of predictors used for differentiation better.

As the DCMD method derives its major improvement from a focus on modelling lower count data and the associated uncertainty, it is necessary to accurately specify an underlying set of mixture components for the low rates. The mixture also has to model low- and high-count data on the same scale, where the density of the latter is often sparse due to sparse observation intervals. Moreover, it is not feasible to apply a transformation to make the data denser due to abundant zeros and the discrete nature of the low counts. However, the weighing of nested candidate models and the suggested heuristic of specifying higher count distributions on a log-linear scale has worked well in our simulations, as it partially mimics the log-transformation commonly applied to such data.

The proposed DCMD method is formulated in a distance-based framework, so it does not include specific mechanisms for variable selection. While different predictors can alternatively be included in the distance sum, the process is not automated. In our case, we used a simple nonparametric Mann-Whitney U test for feature selection, which worked well in the study data. However, more advanced and specialized methods for feature selection can be applied separately for other applications. Additionally, we note that the model is specified to use microbiome site counts, and continuous covariates need to be modelled separately using continuous distributions, while categorical covariates can only be included as dummy variables. These variables will also be treated on the same scale in the distance metric unless specified otherwise.

Despite these drawbacks, we believe that our core contribution, the representation of observations as distributions to reflect uncertainty and the use of distributional distance metrics, will be valuable to anyone analyzing sparse data. This formulation can compensate for some of the disadvantages inherent in distance-based methods to such an extent that it achieved competitive performance with more sophisticated classifiers, as well as specially designed approaches like NSC. The techniques that made DCMD advantageous for classification when data is expected to be sparse, particularly within a distance-based framework, should be considered for improving model performance.

## Conclusion

In this paper, we present a distance-based classification method for microbiome count data. The DCMD approach models the observed data using mixture distributions and calculates *L*^2^-norms for distance-based classification algorithms. The method is specifically designed to accurately model low-count structures, addressing the inherent sparsity by representing each observed count as a distribution, and is demonstrated to have improved performance by simulation studies and two microbiome applications. The importance of accounting for uncertainty in sparse data is emphasized, and the resulting improvements in classification accuracy when using distributions are demonstrated. The performance of the proposed DCMD is competitive to a number of machine learning methods and significantly outperforms other common metrics in distance-based classification models. The consistent and improved performances across a variety of different data structures make this approach a viable alternative for modelling and classification of microbiome count data, particularly within a distance-based framework.

## Supporting information

S1 Text Supporting information for model specification of classifiers. (DOCX)

S1 Table Three-class outcome: the parameter setting for each scenario and the corresponding ZP and mean count for each class over 100 replicates. (DOCX)

S2 Table The nested models of mixture distribution components used in fitting one of the simulated data. (DOCX)

S3 Table Dataset 1—Colorectal Cancer: the predictive performance of the 14 classifiers using the OTUs selected from Mann–Whitney U test on the colorectal cancer data. (DOCX)
