# Supplementary material for: DCMD: Distance-based classification using mixture distributions on microbiome data
Source: PLoS Comput Biol. 2021 Mar 12;17(3):e1008799. doi: 10.1371/journal.pcbi.1008799 (PMC7990174; doi:10.1371/journal.pcbi.1008799)
Supplement: S1 Text — (DOCX) [file pcbi.1008799.s001.docx]

**DCMD: Distance-based Classification Using Mixture Distributions**

Konstantin Shestopaloff^1†^, Mei Dong^1†^, Fan Gao^1^, Wei Xu^1,2*^

1. Dalla Lana School of Public Health, University of Toronto, Toronto, Ontario, Canada

2. Princess Margaret Cancer Centre, University Health Network, Toronto, Ontario, Canada

**Model Specifications of Classifiers**

Distance-based classification including *k*-means and *k*-NN based on Euclidean distance and Manhattan distance and NSC requires us to transform OTU into the Euclidean space. Hence, we normalize the OTU relative abundance to apply to the predictive model. For *k*-means based on Euclidean distance and Manhattan distance, the distance between the new sample and class mean is calculated. The class mean is the average of normalized OTU for each class of the training set. In terms of *k*-NN based on Euclidean distance and Manhattan distance, 10-fold cross-validation (CV) is implemented in the training set to find the optimal *k*. To implement NSC, we use the R package *pamr* [1]. As for the machine learning method, most of them require choosing the optimal tuning parameters. For example, we select the tuning parameter $\lambda$ for LASSO and ridge regression with the minimal error rate from the training set using CV. We fit LASSO and ridge regression on $log(x+1)$ transformed OTU counts using R package *glmnet* [2]. For gradient boosting, the number of trees is selected from 500, 600, 800, 900, and 1000. Other parameters are fixed. R package *gbm* [3] is utilized for gradient boosting. Different to gradient boosting, we choose from 100, 500, or 1000 trees for the random forest. The number of variables selected at a node split is chosen from integers 3 to 8. We use R package *randomforest* [4]. The best combination of the number of trees and the number of variables is returned through 10-fold CV using R package *caret* [5]. For SVM, we choose the optimal result from a linear kernel and a radial kernel, and the linear kernel produces the optimal result for our simulation and real data. R package *e1071* is employed for SVM [6]. All the simulations and real data applications are conducted based on R v.3.5.0.

**References**

1. Hastie, T., R. Tibshirani, Narasimhan, B, Chu, G. pamr: Pam: prediction analysis for microarrays. R package version 1.55. 2014.
2. Friedman J, Hastie T, Tibshirani R. Regularization Paths for Generalized Linear Models via Coordinate Descent. J Stat Softw. 2010;33:1–22.
3. Ridgeway G. Generalized Boosted Models: A guide to the gbm package. :15.
4. Breiman L. Random Forests. Machine Learning. 2001;45:5–32.
5. Kuhn M. Building Predictive Models in R Using the caret Package. Journal of Statistical Software. 2008;28:1–26.
6. Meyer, D., Dimitriadou, E., et al. Package ‘e1071’. The R Journal. 2019.
