# Supplementary material for: DCMD: Distance-based classification using mixture distributions on microbiome data
Source: PLoS Comput Biol. 2021 Mar 12;17(3):e1008799. doi: 10.1371/journal.pcbi.1008799 (PMC7990174; doi:10.1371/journal.pcbi.1008799)
Supplement: S1 Table — (DOCX) [file pcbi.1008799.s002.docx]

Table S1: Three-class outcome: the parameter setting for each scenario and the corresponding ZP and mean count for each class over 100 replicates.

| Scenario | Signal | Sparsity | Class | Size | $\boldsymbol{\alpha}_{\boldsymbol{b}}$ | Mean ZP (SD) | Mean |
| --- | --- | --- | --- | --- | --- | --- | --- |
| 1 | Strong | Low | 1 | 400 | (1.2, 1.8) | 0.37 (0.14) | 6.99 |
|  |  |  | 2 | 400 | (2.0, 2.4) | 0.23 (0.11) | 11.04 |
|  |  |  | 3 | 400 | (2.6, 3.0) | 0.15 (0.09) | 14.83 |
| 2 | Strong | High | 1 | 400 | (0.2, 0.3) | 0.87 (0.06) | 0.85 |
|  |  |  | 2 | 400 | (0.6, 0.7) | 0.67 (0.11) | 2.52 |
|  |  |  | 3 | 400 | (1.0, 1.1) | 0.50 (0.14) | 4.49 |
| 3 | Strong | Very  High | 1 | 400 | (0.1, 0.2) | 0.92 (0.04) | 1.16 |
|  |  |  | 2 | 400 | (0.25, 0.35) | 0.84 (0.07) | 1.17 |
|  |  |  | 3 | 400 | (0.5, 0.6) | 0.72 (0.10) | 1.18 |
| 4 | Null | Low | 1 | 400 | (1.2, 1.8) | 0.37 (0.14) | 10.98 |
|  |  |  | 2 | 400 | (2.0, 2.4) | 0.23 (0.11) | 10.91 |
|  |  |  | 3 | 400 | (2.6, 3.0) | 0.15 (0.09) | 10.97 |
