# Supplementary material for: DCMD: Distance-based classification using mixture distributions on microbiome data
Source: PLoS Comput Biol. 2021 Mar 12;17(3):e1008799. doi: 10.1371/journal.pcbi.1008799 (PMC7990174; doi:10.1371/journal.pcbi.1008799)
Supplement: S2 Table — (DOCX) [file pcbi.1008799.s003.docx]

Table S2: The nested models of mixture distribution components used in fitting one of the simulated data.

| Model/Included | Model 1 | Model 2 | Model 3 | Model 4 | Model 5 |
| --- | --- | --- | --- | --- | --- |
| $\boldsymbol{P}\left( \boldsymbol{X=0} \right)\boldsymbol{=1}$ | X | X | X | X | X |
| $\boldsymbol{\Gamma}\left( \boldsymbol{1, 2} \right)$ | X |  |  |  |  |
| $\boldsymbol{\Gamma}\left( \boldsymbol{1, 1} \right)$ | X | X |  |  |  |
| $\boldsymbol{\Gamma}\left( \boldsymbol{2, 1} \right)$ | X | X | X |  |  |
| $\boldsymbol{\Gamma}\left( \boldsymbol{3, 1} \right)$ | X | X | X | X |  |
| $\boldsymbol{\Gamma}\left( \boldsymbol{4, 1} \right)$ | X | X | X | X | X |
| $\boldsymbol{\Gamma}\left( \boldsymbol{5, 1} \right)$ | X | X | X | X | X |
| $\boldsymbol{\Gamma}\left( \boldsymbol{6, 1} \right)$ | X | X | X | X | X |
| $\boldsymbol{\Gamma}\left( \boldsymbol{7, 1} \right)$ | X | X | X | X | X |
| $\boldsymbol{\Gamma}\left( \boldsymbol{8, 1} \right)$ | X | X | X | X | X |
| $\boldsymbol{\Gamma}\left( \boldsymbol{11, 1} \right)$ | X | X | X | X | X |
| $\boldsymbol{\Gamma}\left( \boldsymbol{16, 1} \right)$ | X | X | X | X | X |
| $\boldsymbol{P}\left( \boldsymbol{X>16} \right)\boldsymbol{=1}$ | X | X | X | X | X |
