# Supplementary material for: DCMD: Distance-based classification using mixture distributions on microbiome data
Source: PLoS Comput Biol. 2021 Mar 12;17(3):e1008799. doi: 10.1371/journal.pcbi.1008799 (PMC7990174; doi:10.1371/journal.pcbi.1008799)
Supplement: S3 Table — (DOCX) [file pcbi.1008799.s004.docx]

Table S3: Dataset 1 - Colorectal Cancer: the predictive performance of the 14 classifiers using the OTUs selected from Mann–Whitney U test on the colorectal cancer data.

| Method | Accuracy | Precision | Recall | F1 score |
| --- | --- | --- | --- | --- |
| *k*-means-D-*L*^2^ | **0.67** | 0.66 | **0.69** | **0.67** |
| *k*-means-CC-*L*^2^ | 0.63 | 0.62 | **0.67** | **0.65** |
| *k*-means-Euclidean | 0.62 | 0.62 | 0.60 | 0.61 |
| *k*-means-Manhattan | 0.65 | 0.66 | 0.60 | 0.63 |
| *k*-NN-D-*L*^2^ | 0.65 | **0.77** | 0.43 | 0.55 |
| *k*-NN-CC-*L*^2^ | 0.63 | 0.65 | 0.57 | 0.61 |
| *k*-NN-Euclidean | 0.63 | 0.66 | 0.55 | 0.60 |
| *k*-NN-Manhattan | 0.61 | 0.69 | 0.40 | 0.50 |
| NSC | **0.67** | **0.72** | 0.56 | 0.63 |
| RF | 0.62 | 0.61 | 0.63 | 0.62 |
| GB | 0.60 | 0.60 | 0.63 | 0.61 |
| LASSO | 0.62 | 0.62 | 0.64 | 0.63 |
| RR | 0.64 | 0.63 | **0.67** | **0.65** |
| SVM | **0.67** | 0.67 | 0.67 | **0.67** |
